# Oral Findings in Male Prisoners: A Systematic Review

**DOI:** 10.3390/jcm13061736

**Published:** 2024-03-17

**Authors:** Rafał Korkosz, Agata Trzcionka, Tomasz Hildebrandt, Maksymilian Kiełbratowski, Anna Kuśka-Kiełbratowska, Mansur Rahnama, Marta Tanasiewicz

**Affiliations:** 1 Department of Conservative Dentistry with Endodontics, Faculty of Medical Sciences in Zabrze, Medical University of Silesia, Plac Akademicki 17, 41-902 Bytom, Poland; rkorkosz@sum.edu.pl (R.K.); thildebrandt@sum.edu.pl (T.H.); mkielbratowski@sum.edu.pl (M.K.); martatanasiewicz@sum.edu.pl (M.T.); 2 Department of Periodontal Diseases and Oral Mucosa Diseases, Faculty of Medical Sciences in Zabrze, Medical University of Silesia, pl. Traugutta 2, 41-800 Zabrze, Poland; anna.kuska-kielbratowska@sum.edu.pl; 3 Department of Dental Surgery, Medical University of Lublin, Witolda Chodźki 6, 20-093 Lublin, Poland; mansur.rahnama@umlub.pl

**Keywords:** systematic review, oral status, oral hygiene, prisoners, inmates, treatment needs, databases, Web of Science, SCOPUS

## Abstract

**Background:** Modern penitentiary systems attach great importance, at least in the area of formal and codified arrangements, to providing inmates with access to health care and rehabilitation. The aim of our study was to analyze the Web of Science (WoS) and SCOPUS medical databases in order to search for and evaluate the available literature discussing the oral status and dental treatment needs of adult male prisoners. **Methods:** The following terms were used: prisoners or inmates; oral health, oral status; periodontal status, periodontal disease; oral hygiene; caries; mucosa; and saliva. The studies were screened based on their title and abstract according to the PICO (population, intervention, control, and outcome) criteria. The research protocol was prepared on the basis of the 2020 PRISMA guidelines and was not registered. The available literature discussing the oral status and dental treatment needs of adult imprisoned patients was analyzed. The inclusion criteria were as follows: articles published in English between 1 January 2012 and 2022; articles discussing the oral cavity status of adult inmates over 18 years old (hard tissues, periodontal status, saliva, mucosa condition, or oral hygiene); articles with a full text available; and articles that were assessed as satisfactory according to the Newcastle–Ottawa Scale. **Results**: A total of 934 articles were identified, out of which 9 were included in the systematic review. Two articles discussed the oral condition of prisoners in Europe (Russia and Finland), four examined prisoners in Asia (three in India and one in Saudi Arabia), two examined prisoners in Africa (Nigeria), and one examined prisoners in the Americas (Brazil). **Conclusions:** The oral status of prisoners has been widely discussed in the available literature. Among inmates, a higher frequency of both caries and periodontal disease along with poorer oral hygiene were observed. It can be concluded that inmates should have access to specialized treatment from periodontists and endodontists.

## 1. Introduction

Contemporary penitentiary systems pay significant attention, at least in terms of formal establishments, to providing inmates with appropriate health services and resocialization. The number of prisoners is increasing worldwide; however, there are differences between continents and within continents. According to statistical data, most prisoners are in the United States (nearly 2.1 million people in prisons), followed by China, Brazil, India, and the Russian Federation. The number of prisoners per 100,000 people in the world varies significantly, with 37 in Japan, 70 in Germany, 91 in Austria, 129 in Estonia, 190 in Poland, 253 in Georgia, 347 in Turkey, 580 in Rwanda, and 629 in the United States [1]. In the Polish penitentiary system, there are 87 prisons and 34 external units of different types, which, together, provide a capacity for 83,000 prisoners. There are 81,000 inmates, and 30,000 people have been sentenced and are waiting for imprisonment. Annually, 90,000 people are released from prisons in Poland, which averages to 7650 per month, while 7400 prisoners are admitted to prison [2].

Oral health is an integrated part of general well-being. Many different factors may influence oral health, such as a person’s social status, profession, education, ability to write or read, and nutritional status. The identification of unique social groups as well as the assessment of their health problems is one of the tasks of public health. Prisoners are considered a specific population due to their movement restrictions. Their living conditions are very similar, and their access to health care is uniform [3,4,5]. It needs to be highlighted that inmates are exposed to many threats related to access to illegal substances, such as drugs or nicotine. They often suffer from self-destructive patterns and personality or mental disorders. Prisoners are frequently diagnosed with anxiety, depression, bipolar disorder, psychosis, psychopathy, schizophrenia, and personality disorders [6]. Imprisonment may lead to a diminution in inmates’ QoL (quality of life). This process is a result of the deprivation of many important needs—above all, the need for autonomy and freedom and the need for social contact. Prisoners are deprived of good material conditions and a high economic status—factors that increase QoL [6]. Health services for inmates are provided in health centers for prisoners, which are a part of the organizational structures of penitentiary systems and include outpatient clinics with infirmaries, prison hospitals, diagnostic laboratories, dental offices, and rehabilitation or physical therapy offices. The penitentiary health system is organized and supervised by the chief prison doctor with a group of specialists on the Central Board of the Prison Service. A chief prison doctor is employed in each of the 15 District Inspectorates of the Prison Service [2]. Due to the limited number of dental professionals and resources in most prisons, especially in developing countries, it is difficult for prisoners to receive routine and appropriate oral health care. Hence, the burden of oral diseases is substantially higher among this vulnerable group when compared to the general population [4]. Despite such an organization, the health care system for prisoners is inadequate, and in the field of dentistry, it is implemented in external locations, which influences its availability. Several studies have reported the oral health status of prison populations and have shown a high prevalence of dental caries, oro-mucosal lesions, precancerous lesions, poor periodontal status, and missing teeth [4,7,8,9,10]. An extensive review of the literature revealed that the few studies carried out in a prison setting showed a higher prevalence of dental caries and periodontal diseases [5,7,8,9,10]. The aim of our study was to analyze the Web of Science (WoS) and SCOPUS medical databases in order to search for and evaluate the available literature discussing the oral status and dental treatment needs of adult male prisoners. On the basis of the included studies, the authors aimed to gather information on the oral status of male inmates.

## 2. Materials and Methods

**Figure 1:**
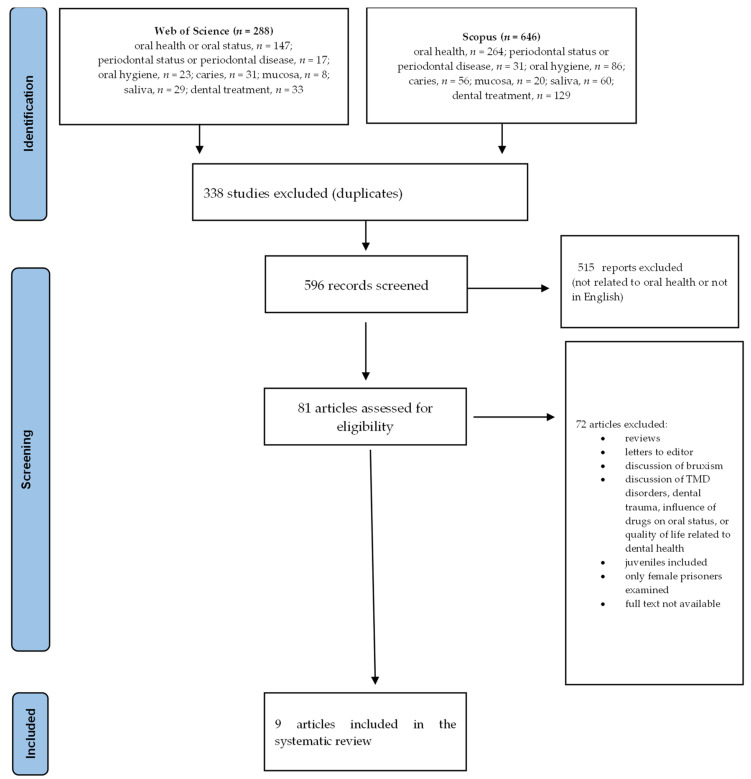
The study selection process, presented with the usage of a PRISMA flowchart.

The research protocol was prepared on the basis of the 2020 PRISMA guidelines and was not registered (Figure 1) [11]. The available literature discussing the oral status and dental treatment needs of adult imprisoned patients was analyzed. The Web of Science (WOS) and SCOPUS databases were searched. The inclusion and exclusion criteria were as follows:

Inclusion criteria: articles published in English between 1 January 2012 and 2022; articles discussing the oral cavity status of adult inmates over 18 years old (hard tissues, periodontal status, saliva, mucosa condition, or oral hygiene); articles with a full text available; and articles that were assessed as satisfactory according to the Newcastle–Ottawa Scale.

Exclusion criteria: case reports and reviews where the full text was not available, and studies discussing the oral status of inmates younger than 18 years old.

MeSH (medical subject heading) indexation was used to choose the appropriate terms for searching the databases. However, based on our own experience in the field of epidemic studies, some keywords that were not indexed in the MeSH were also included. As a consequence, the following sets of keywords were used: prisoners OR inmates; oral health OR oral status; periodontal status OR periodontal disease; oral hygiene; caries; mucosa; and saliva.

The publications were screened based on their title and abstract using the PICO (population, intervention, control, and outcome) criteria. Following the PICO criteria, the following question was prepared: Are adult male imprisoned patients at an increased risk of developing oral pathologies, such as dental caries, periodontal diseases, mucosa pathologies, or poor oral hygiene, compared with adult male non-imprisoned patients of the same age?

Two independent and knowledgeable researchers (A.T. and R.K.) performed the article selection on 14 September 2022. The Cohen’s kappa value between the researchers was 0.41, so their agreement was assessed as moderate [12]. Cohen’s kappa values can be interpreted as follows:

- <0.00—poor;
- 0.00–0.20—slight;
- 0.21–0.40—fair;
- 0.41–0.60—moderate;
- 0.61–0.80—substantial;
- 0.81–1.00—almost perfect.

Substantive analysis

One researcher (A.T.) screened the studies included in the review and gathered data regarding the year and country of each study’s publication and the characteristics, tooth condition, periodontal tissue, and mucosa and oral hygiene status of the study participants. The obtained information was examined by another coauthor (M.T.) to eliminate the risk of bias.

Quality assessment

The included manuscripts were assessed using the Newcastle–Ottawa quality assessment scale in the following domains: selection (SD), comparability (CD), and outcome (OD). The maximum score was 10, and in these domains, the following scores were assigned: selection—5, comparability—2, and outcome—3. The interpretation of the Newcastle–Ottawa quality scale was as follows:

- 9–10 points: very good quality;
- 7–8 points: good quality;
- 5–6 points: satisfactory quality;
- 0–4 points: unsatisfactory quality [12,13].

This task was performed separately by two authors (A.T. and R.K.), and if a discrepancy occurred, the result was decided by a third author (M.R.).

## 3. Results

### 3.1. Quality Assessment of Studies Included in the Systematic Review

A quality assessment of the six studies included in the review was performed using the Newcastle–Ottawa scale. The procedure was performed by two coauthors independently, and in the case of a discrepancy, a third author made a decision (Table 1).

The quality of the articles included in the review ranged from satisfactory to very good. Only one article was assessed as very good.

### 3.2. Characteristics of the Included Studies

Searching the Web of Science and Scopus databases resulted in the identification of 934 articles. After the duplicates were eliminated, 596 publications were left (Figure 1). In the first stage of the analysis, articles not related to dentistry and those published in any language other than English were rejected. Finally, nine manuscripts were analyzed (Table 2). Two articles discussed the oral condition of prisoners in Europe (Russia and Finland) [10,14], four discussed prisoners in Asia (three in India and one in Saudi Arabia) [7,9,15,16], two discussed prisoners in Africa (Nigeria) [17,18], and one discussed prisoners in America (Brazil) [4]. The hard tissue condition was assessed in all of the included studies (with the use of DMFT index 0214870 (decayed, missing, and filled permanent teeth)), the periodontal status was examined in five of them, and only one study described the oral hygiene of the prisoners. Table 2 presents a summary of the characteristics of the studies included in the review.

### 3.3. Oral Findings in Male Inmates on the Basis of the Studies Included in the Review

The data from the included studies regarding the hard tissue conditions, periodontal status, mucosal status, and oral hygiene of the prisoners are presented in Table 3, Table 4 and Table 5.

The authors of the included studies that investigated tooth conditions and the prevalence of caries agreed that the frequency of caries in the prisoners was high: George et al. [9]—54.2% of females and 58.2% of males; Radebe et al. [18]—64.34% of the participants; Bukhari et al. [7]—90.2% of the prisoners; and Reddy et al. [15]—97.5%.

**Table 3:** Data regarding hard tissue conditions.

Reference Number	Hard Tissue Conditions
[15]	Prevalence of caries—97.5%; Mean decayed, missing, or filled teeth (DMFT)—5.26; One or more decayed (D) teeth—92.5%; One or more missing (M) teeth—57.1%; One or more filled (F) teeth—24.6%.
[7]	Percentages of prisoners with decayed (D) (90.2%) or missing (M) (80.5%) teeth were higher than those of the control group (D = 57%, M = 60.8%); Percentage of prisoners with filled (F) teeth was 31.7%, while for the controls, it was 50.6%; Median DMF was higher in prisoners than in controls (M = 8 vs. M = 5, *p* = 0.001).
[8]	Mean DMFT index value was 19.72; Mean values of each component were as follows: D (11.06 ± 5.37), M (7.20 ± 7.23), and F (1.46 ± 2.45).
[9]	58.2% of males and 54.2% of females had decayed teeth; About 4.1% of males and 2.9% of females had filled teeth; Mean decayed, missing, and filled teeth index was 5.1 and 3.9 for female and male prisoners, respectively.
[10]	Mean DMFT and the values of its components in the entire group were as follows: DMFT = 16.8 (±8.9), D = 5.0 (±5.11), F = 6.9 (±5.15), and M = 4.7 (±6.01); In prisoners younger than 30 y., D = 4.8 (±5.76) and DMFT = 12.4 (±7.5); In prisoners older than 30 y., D = 5.1 (±4.80) and DMFT = 19.0 (±8.80).
[14]	Mean DMFT was 14.86 (±0.26), D = 6.48 (±2.18), M = 2.92 (±1.24), and F = 4.24 (±1.29).
[16]	Total DMFT score was 180 and average DMFT was 1.37 for the total prison population; Percentages of prisoners with decayed, missing, or filled teeth were as follows: 64.9%, 61.1%, and 7.7%, respectively.
[17]	67% of the prisoners had decayed teeth or teeth missing as a result of caries.
[18]	Mean DMFT scores were as follows: overall, 5.92 (+4.65); 18 to 29 years, 4.14 (+3.49); 30 to 39 years, 6.17 (+4.19); 40 to 49 years, 9.08 (+5.38); and older than 50 years, 11.31 (+6.30). A statistically significant relationship was found between DMFT and age (*p*-value: 0.000). Decayed teeth were found in 64.34% of the participants, 71.85% recorded missing teeth, and filled teeth (FT) were noted in only 8.04% of the study sample.

Four of the studies included in this review discussed the results of the CPI (community periodontal index) [10,11,15,17]. In two of the studies, most of the patients were diagnosed with CPI 2 or 3 [10,14]. Reddy et al. found that, in most of the participants, a CPI score of 2 (39.3%) or either 3 or 4 (48.6%) was observed [15]. Only one study—Valnionpää et al.—calculated the bleeding on probing (BOP) index. They noted bleeding on probing in 4–6 sextans in 88% of the entire examined group, in 93.9% of the prisoners under 30 years old, and in 85.1% of those aged 30 and over.

**Table 4:** Data regarding periodontal tissue conditions.

Reference Number	Periodontal Status
[15]	CPI (community periodontal index) score of 2 in 39.3% of prisoners and 3 or 4 in 48.6% of prisoners; LOA score of 1 or 2 in 30.1% of prisoners and 4 in 1.7% of prisoners.
[10]	Percentages of CPI in entire group were as follows: CPI1—7%, CPI2—52%, CPI3—34%, and CPI4—7%; In those under 30, the scores were as follows: CPI1—2.9%, CPI2—67.7%, CPI3—26.5%, and CPI4—2.9%; In those 30 and over, the scores were as follows: CPI1—9.1, CPI2—49.9%, CPI3—25.3%, and CPI4—9.1%; The percentage of prisoners with BOP (bleeding on probing) was as follows: 0–1 sextans (A)—6%, 2–3 sextans (B)—6%, and 4–6 sextans (C)—88%; in those under 30, the percentages were as follows: A—3%, B—3%, and C—93.9%; in those 30 and over, the percentages were as follows: A—7.5%, B—7.5%, and C—85.1%.
[14]	Data were presented with regard to sex and time of incarceration: Males <5 years: CPI0—0, CPI1—5%, CPI2—36.3%, CPI3—33.7%, and CPI4—20%; Males >5 years: CPI0—0, CPI1—0, CPI2—22.1%, CPI3—33.7%, and CPI4—7%; Females <5 years: CPI0—0, CPI1—8%, CPI2—46.7%, CPI3—29.3%, and CPI4—12%; Females >5 years: CPI0—o, CPI1—1.8%, CPI2—29.1%, CPI3—32.7%, and CPI4—23.7%.
[17]	CPI0 was observed in 5.2% of participants, and CPI1–4 was observed in 94.8% of them.

The oral hygiene in prisoners was assessed only by the researchers from Russia [14]. They calculated the s-OHI (simplified oral hygiene index) with regard to sex and the time of incarceration and obtained the following results: M < 5 = 2.42 (±0.03), M > 5 = 3.11 (±0.03), F < 5 = 2.53 (±0.03), and F > 5 = 2.62 (±0.03), where M—males and F—females.

Additionally, one study presented data collected from an analysis of the medical history based on the International Classification of Diseases, 10th revision—Clinical Modification (ICD-10CM) and proved that the prevalence of specific diseases was as follows: caries—92.4%, excessive teeth attrition—56.4%, calculus—89.5%, pulp and periapical tissue diseases—87.9%, gingivitis—33.1%, periodontitis—55.7%, gingival recession—26.9%, cheilitis—64.9%, complete loss of teeth—7.5%, and partial loss of teeth—81.9% [14].

Two studies gathered information on the mucosal pathologies observed in inmates [14,15]. According to their results, the most frequent diseases affecting the oral mucosa of prisoners were as follows: oral submucous fibrosis, ulcers, leukoplakia, cheek and lip biting, leukokeratosis nicotina palati, hyperplasia of mucosa, tongue diseases, and candidiasis (Table 5). The researchers from Russia collected data on the basis of medical history analyses.

**Table 5:** Pathologies regarding mucosa observed in inmates.

Reference Number	Pathology
[15]	Oral submucous fibrosis—9.9%; Ulcers—7%; Leukoplakia—1.1%.
[14]	Cheek and lip biting—53.1%; Leukoplakia—8.2%; Leukokeratosis nicotina palati—28.8%; Hyperplasia of mucosa—6.8%; Tongue diseases—21.9%; Candidiasis—4.9%.

## 4. Discussion

The scientific literature contains extensive research on the health and functioning of people in isolation for various reasons. The state of the health and mental well-being of inmates has been analyzed in many extensive studies. The subject of health focuses on people in extremely poor health due to poverty, marginalization, or multimorbidity. The authors of a study investigating this topic aimed to review the morbidity and mortality data on four overlapping populations who experience considerable social exclusion: homeless populations, individuals with substance use disorders, sex workers, and imprisoned individuals [19]. Another study described self-harm, which is a leading cause of morbidity in prisoners [20]. However, these publications were not included in our selection due to the lack of an analysis of the state of the oral cavity. When the selection process was completed, articles that included studies from all over the world were included. The majority of the manuscripts included in this review originated from Asia and Africa, and only two were conducted in Europe; this was the result of a larger number of inmates in non-European regions. A standardized review of the literature was performed by our team, which enabled the studies in the literature to be reduced to nine main articles, the results of which have been discussed in this review. However, only one out of the chosen studies was correlated with our decision to focus on oral status analyses only in male inmates [17]. It needs to be underlined that, even though the rest of the articles discussed the oral condition of prisoners belonging to both sexes, this was considered inappropriate due to the significant disproportion in the number of men and women in all of the cited studies.

All the studies included in this review analyzed the condition of inmate dentition. The cited research demonstrated that there are high treatment needs for prison inmates in terms of conservative treatment. Reddy et al. [15] observed that 92.5% of inmates were diagnosed with one or more teeth with caries; their observations were similar to those of Bukhari et al. [7], who reported 90.2% of inmates having caries. Lower numbers of prisoners with caries were observed by George et al. (58.2% of males and 54.2% of females) [9], Rinki et al. (64.9%) [16], Akaji et al. (67%) [17], and Rabede (64.34%) [18]. However, it should be pointed out that more than half of the participants in these studies were diagnosed with caries. 

Bukhari et al. [7] compared the DMF index and its components between a group of prisoners and a control group composed of people living in freedom. They observed a higher percentage of carious teeth and missing teeth in prisoners when compared to the control group; however, the percentage of filled teeth was lower in the prisoners than in the people living in freedom. They noted higher DMF index values in the examined group. Similar results were obtained by Korkosz et al. [21]; they observed that the D and M values were significantly higher in the examined group (*p* < 0.001) and found the following values: D—7.0 in the examined group and 5.0 in the control group, and M—5.0 in the examined group and 2.5 in the control group. The value of F was significantly higher in the control group—5.5—than in the examined group—2.0 (*p* < 0.001). The cited data may indicate the unavailability of dental treatments in prisons or that their availability is unsatisfactory when taking into account the treatment needs.

Only the researchers from Russia assessed the oral hygiene of inmates [14]. They observed the following values of the s-OHI (simplified oral hygiene index) with regard to an inmate’s time spent in the prison: M < 5 = 2.42 (±0.03) and M > 5 = 3.11 (±0.03). Although the results obtained by them were interpreted as representing satisfactory hygiene, it was observed that the inmates who spent more than five years in the prison experienced an increasing average value of their oral hygiene index, which suggests that oral hygiene negligence increases with the length of imprisonment.

In four of the articles included in this review, the analysis of the periodontal status in inmates was performed using the CPI index. Two groups of authors noted that there were definitely more people with CPI 2 or CPI 3. Vainionpaa et al. [10] assessed the CPI in prisoners and concluded that people were most frequently classified as CPI 2 or 3, with CPI2—52% and CPI3—34%. Additionally, the authors assessed the periodontal status with regard to the age of the participants, and again, they observed that more inmates were classified into the two groups already mentioned: the under 30 group: CPI2—67.7% and CPI3—26.5%, and the 30 and over group: CPI1—9.1, CPI2—49.9%, and CPI3—25.3%. Kondratyev et al. [14] determined the percentages of men classified with CPI 2 or CPI 3 with regard to their length of imprisonment: males <5 years: CPI2—36.3% and CPI3—33.7%; males >5 years: CPI2—22.1% and CPI3—33.7%. Similar results were presented by Reddy et al. [15], who reported a CPI score of 2 in 39.3% of prisoners and a CPI score of 3 or 4 in 48.6% of prisoners, and Akaji et al. [17], who reported CPI1-4 in 94.8% of prisoners. In the analyzed group of prisoners, the following pathologies were observed: dental pockets of 3 mm or 3.5–5.5 mm, calculus or dental plaque above or below the gum level, overhanging restorations, and bleeding on probing [22]. It was concluded that inmates should be given the opportunity to receive specialistic treatment from periodontologists. 

The results regarding periodontal status should undoubtedly be correlated with the Russian authors’ observations on oral hygiene, due to the fact that the hygiene level and the quality of hygienic procedures are two of the main factors in the etiology of periodontal diseases. In our research [21], due to the fact that an analysis of the patients’ medical history and X-rays was conducted, it was possible to evaluate the condition of the periapical tissues of endodontically treated teeth, marginal periodontium, and horizontal and vertical atrophy of the bone with respect to the condition of the teeth after endodontic therapy. We observed that the D and M values were significantly higher in the examined group (*p* < 0.001); the values for D were 7.0 in the examined group and 5.0 in the control group, and the values for M were 5.0 in the examined group and 2.5 in the control group. Our own research proved that there was no statistically significant difference between the groups in the presence of periapical changes in endodontically treated teeth (*p* = 0.389). This could lead to the conclusion that the implemented treatment was of a comparable effectiveness for both the prisoners and the control group. In the control group, for which the frequency of appointments at a dental office was significantly higher, the decision to perform a re-endo treatment was also significantly more frequent. High-quality, careful, and frequent dental check-ups can result in the identification of particular needs for treatment. The number of teeth that qualified as needing to be endodontically treated for a second time was significantly higher in the control group (*p* = 0.001). The odds ratio proved that the examined group was characterized by an over 4.2-times higher probability of periapical changes occurring in the teeth, with no previous endodontic treatment, when compared to the control group (*p* < 0.001). The odds ratio proved that the probability for the occurrence of horizontal atrophy in the maxillary and mandibular alveolar processes in the examined group was over three times higher than in the control group (*p* < 0.001). The differences in the results for vertical atrophy in the alveolar process showed a tendency toward statistical significance. Vertical atrophy was observed more often in the examined group and was present in 27 prisoners and absent in 59. In the control group, vertical atrophy was diagnosed in 59 people and lacking in 87 [21]. Unfortunately, despite a very careful analysis of the literature, we could not find any manuscripts discussing the endodontic treatment or atrophy of the alveolar processes of the mandibula and maxilla.

It is crucial to ask whether there is an adequate availability of oral hygiene agents such as dental floss, oral rinses, and irrigators that could decrease the problem of periodontal diseases. There is a need to conduct research on the awareness of inmates on the influence of proper oral hygiene procedures and their impact on caries and periodontal disease occurrence.

Two articles discussed the mucosa status. Reddy et al. [15] observed a very random occurrence of oral submucous fibrosis among 9.9% of prisoners, ulcers among 7%, and leukoplakia among 1.1%. Kondratyev et al. [14] noted the following on the basis of a medical history analysis: cheek and lip biting—53.1%, leukoplakia—8.2%, leukokeratosis nicotina palati—28.8%, hyperplasia of mucosa—6.8%, and tongue diseases—21.9%. Most of these pathologies are connected to cigarette smoking, which is a substantial problem in prisons, as previously mentioned [6]. This may indicate an insufficient awareness of the prisoners of the influence of cigarette smoking on their oral health. It is important to analyze this problem further and educate inmates.

## 5. Conclusions

The oral status of prisoners has been widely discussed in the available literature. This group of patients provides a good source of material for research due to the fact that they live in a comparable environment. The majority of the articles were from Asia, which may have been caused by the higher number of prisons located there. However, many significant and detailed studies have been performed on large groups of inmates that did not meet the criteria of this review due to a lack of an analysis on the state of the oral cavity.

A higher frequency of caries and periodontal diseases and a lower level of hygiene were observed in male prisoners. It can be concluded that inmates should be provided with access to specialistic treatment from periodontologists and endodontics.

None of the articles in this study discussed the problem of periapical tissue conditions after an endodontic treatment, nor the atrophy of the alveolar processes of the mandibula and maxilla, which could be correlated according to the available imaging diagnostics.

## Figures and Tables

**Table 1:** Results of quality assessment using the Newcastle–Ottawa scale.

Reference Number	Selection	Comparability	Outcome	Quality
15	3	2	3	GOOD
7	3	1	2	SATISFACTORY
8	2	0	3	SATISFACTORY
18	3	1	3	GOOD
9	4	1	3	GOOD
10	2	1	3	SATISFACTORY
14	2	2	3	GOOD
16	4	2	3	VERY GOOD
17	4	1	0	SATISFACTORY

**Table 2:** Characteristics of studies included in review.

Author, Title, and Year of Publication	Country	Participants/Materials	Statistical Analysis
Reddy et al., “A survey on oral health status and treatment needs of life-imprisoned inmates in central jails of Karnataka, India”, 2012 [15]	India	800 prisoners; 722 males and 78 females	Arithmetic mean, standard deviation, chi-squared test, analysis of variance (ANOVA), and contingency coefficient. The Statistical Package for Social Sciences (SPSS) ver. 16 was used.
Bukhari et al., “Oral health amongst male inmates in Sudi prisons compared with that of a sample of the general male population”, 2017 [7]	Saudi Arabia	Study group: 82 male prisoners Control group: 79 males	Chi-squared test, Mann–Whitney U test, Spearman’s rho, binary logistic regression, odds ratio, and confidence intervals. Analyses were performed using SPSS ver. 17.
Cavalcanti et al., “Dental caries experience and use of dental services among Brazilian prisoners”, 2014 [8]	Brazil	127 male prisoners	Pearson’s chi-squared test, Fischer’s exact test, and Kruskal–Wallis test. SPSS ver. 17 was used.
Radebe et al., “Investigating dental caries rates amongst sentenced prisoners in KwaZulu-Natal, South Africa”, 2020 [18]	South Africa	373 prisoners; 333 males and 40 females	Standard deviation, frequency distribution, measures of central tendency, and Pearson’s chi-squared test. SPSS ver. 24 was used.
George et al., “Dental caries status of inmates in central prison, Chennai, Tamil Nadu, India”, 2015 [9]	India	1060 inmates; 1025 males and 35 females	Independent *t*-test. Analysis was performed using IBM SPSS ver. 16.
Valnionpää et al.,” Oral health and oral health-related habits of Finnish prisoners”, 2017 [10]	Finland	100 prisoners; 89 males and 11 females	Kappa value, means, minimum and maximum values, frequencies and distributions, Pearson’s chi-squared test, and Fischer’s exact test. Analyses were performed using SPSS ver. 22 (Chicago, IL, USA).
Kondratyev et al., “Prevalence of oral diseases and the assessment of the simplified oral hygiene, decayed-missing-filling and community periodontal indices among inmates of the Russian Federation”, 2019 [14]	Russia	305 inmates; 175 males and 130 females	Pearson’s correlation coefficient and *t*-tests. SPSS ver. 16 was used.
Rinki et al., “Prevalence of dental caries among prisoners of central jail, Jodhpur city, Rajasthan, India”, 2014 [16]	India	131 male prisoners	Descriptive statistics and chi-squared test.
Akaji et al., “Oral health status of a sample of prisoners in Enugu: a disadvantaged population”, 2014 [17]	Nigeria	230 inmates; 224 males and 6 females	Percentages, standard deviation, and Spearman’s correlation rho. Data were analyzed using SPSS ver. 15 (Chicago, IL) and Graph Pad Prism for Windows, ver. 5 (San Diego, California).
